# Cases Illustrating the Use of Iodide of Potassium in the Cerebral Affections of Children

**Published:** 1854-02

**Authors:** Sanford B. Hunt


ART. II.—*Cases illustrating the use of Iodide of Potassium in the Cerebral Affections of Children.* By SANFORD B. HUNT, M. D.

If we assume that chronic hydrocephalus includes only that class of cases when the disease comes on slowly, and is accompanied by tubercular deposit upon the brain or its membranes, the cases following will not come under that designation. In all cases of hydrocephalus occurring in strumous children, or at the close of exhausting illnesses, it has been my fortune to witness a fatal result.

So universally has this held true, that it was common with the older authors to assert, that no case of dropsy of the brain had ever recovered; and that all such reported recoveries were cases of simulated cerebral disease, dependent on irritation elsewhere.

This opinion owes its origin to the fact, that no distinction was made between scrofulous hydrocephalus (tubercular meningitis) and that form produced by the retrocession of tumors, the translation of disease, or by idiopathic cerebritis. This distinction is now widely known, and it is freely admitted that cases of this latter kind may often recover.

It is still, however, a dangerous, and, in a large proportion of cases, a fatal disease. Coming on, as it often does, very suddenly, but little time is furnished for the exhibition of remedies. The prognosis must depend almost entirely on the amount of effusion, and particularly the degree in which the action of the heart and lungs are influenced thereby. I have somewhere seen applied to this sudden form of effusion, the name of "water-stroke," and consider it a very expressive designation for that sudden coming on of symptoms of effusion, which so rapidly destroys life.

But "water-stroke" is not confined to the simple or non-malignant form of hydrocephalus. I have seen it in one case of tubercular meningitis, occurring with a rapidity which merited the name. A little lad, aged seven years, of remarkable personal beauty, and possessing that precocity of intellect and mildness of disposition common to undue cerebral development, had for thirty-six hours, during which I attended him, the symptoms of mild remittent fever. He made no complaint of his head. I visited him at four in the afternoon, and found him sitting on a lounge, perspiring freely, having just had a smart fever. He took some light food during my visit. Two hours after, having been playful in the mean time, he asked for water in a strangely altered tone of voice. When it was brought him he was unable to drink, having already become insensible. I saw him at once. He was comatose, with irregular pulse and respiration, and his right side evidently paralyzed. In this condition he lived twenty-four hours, having some slight convulsions of the left side. I learned subsequently, that my little patient had complained to a neighbor of headache, but he had never mentioned it at home. No autopsy was had, but from his peculiar organization, and from scrofulous difficulties for which I had previously treated him, I have no doubt of the tubercular cause of his sudden death.

I have mentioned this case, only as an illustration of the marked form of tubercular meningitis.

It is doubtless true that many cases of simple effusion may terminate with equal rapidity. It is known that after certain eruptive diseases involving temporary albuminuria, effusions of serum occur very rapidly in the serous cavities. The thorax and abdomen are the more usual localities of these effusions; but it is at least probable, that the brain may likewise be their seat. The cases reported in this Journal two years since by Dr. Flint, though not verified sufficiently by post-mortem appearances, can hardly be explained on any other supposition. I have seen such deaths in several instances. In one case a child had recovered from measles without any treatment, and was standing at six o'clock, P. M., at the window, looking out; suddenly it had difficult respiration, and became at once insensible. I saw her at 11, P. M. She was moribund, the pulse was regular, while the respiration occurred only at rare intervals.

I have once attributed a sudden death to a similar cause. A man, aged

50 years, had had neuralgia of the fifth pair, following an insensibility of a few hours, of which I know nothing save the fact. While convalescing, but still suffering much from neuralgia, he died very suddenly before a physician could be obtained. There is, in this case, room to doubt as to whether it was not true apoplexy.

In other cases, however, I have had reason to predicate the manner of death, from the existence of neuralgic symptoms with great organic disease, and debility. I once predicted to the friends, that a lady suffering from tubercle would die in this way. Six weeks after she complained of pain in the nuchæ, and the side of the head, and died almost immediately.

I could mention other cases of this kind, but they are somewhat foreign to the intention of this paper, and all of them lack the verification of an autopsy.

The cases which I shall now describe, will present some features in common with these fatal cases. In two of them there was a previous zymotic disease, of such form as to be readily transferable by metastisis. In the third there had been a cutaneous eruption.

Case I.—A. C., a boy, aged twenty months, had cynauche parotidea, leaving the gland swollen and tender. He did not seem much ill, until, after several hours of fretfulness, his voice assumed that peculiar shrill and piercing tone known as the *cri hydrencephaliqne.* I found him soon after with head symptoms. The head was hot, the pupil of the eye contracted, and but slightly sensible to light; he lay moaning and tossing his hands upward.

As his bowels had been previously constipated, I prescribed an aloetic enema, a warm bath, cold to the head, and iodide of potassium, gr. j, every two hours. The symptoms did not vary much for two succeeding days. The treatment was continued unvaryingly, a warm bath being given every six hours, while the aloetic enema was administered twice daily. No medicine was given by the mouth except the iod. pot. A profuse secretion of urine followed, and on the third day there was considerable amendment. He passed through a tedious and fretful convalescence. Before his illness he had walked with great firmness, but now he seemed to have lost all control of his limbs, and was in a semi-paralytic condition. This continued for two months, when, as warm weather came on, I ordered him to be exposed to the direct rays of the sun, in a simple chemise, and to be allowed to roll in the fresh earth of the garden. He soon became healthy under the influence of these natural tonics.

The two following cases I will transcribe nearly verbatim from the daily record, made during the progress of the cases:

CASE II.—22d Jan., A. F., aged 5 years, is lively in the morning, but grows sleepy and stupid toward noon. He has had a pustular eruption on the surface, which, within a day or two, has disappeared. Moans in his sleep, throwing his hands to his head, which sweats profusely; lies with one hand pressed against the left temple. Extremities cold and dry; head hot. Strawberry tongue; bowels costive; no appetite.

*Treatment.* Cold to the head, draughts to the feet and wrists, warm bath.

℞ Calomel, grs. v, follow with castor-oil and spts. terebinth.

23*d*. Cathartic operated freely with a greenish stool. He appears lively and playful.

Continue the revulsive treatment.

℞ Hg. biniodid, gr. j.
Iod. potass., grs. xx.
Aqua, ℥j.

Take 10 drops every three hours.

24*th*. Appearance the same as yesterday morning. During last night was delirious, and complained of his head. Gave an infusion of pink and senna, as he was subject to worms, but without effect.

25*th*. Morning. Had a restless night. There is a profuse discharge from the nostril. Gave a cathartic, which operated freely.

Evening. Head is very hot; hands cool and husky; but one discharge of urine in twenty-four hours. The pupil of the eye vacillates, and is on the whole somewhat dilated. Pulse intermits occasionally.

℞ Emp. Vesicat to the nuchæ. Substitute 3 grs. of iod. potass. for the solution of the biniodide, which has been given every three hours. Give two warm baths during the night, and keep a bladder of snow upon his head.

26*th*. Sleepy; has had a very disturbed night. Had two urinary discharges this morning. Pupil of the eye dilated. Countenance fatuitous. Pulse regular, slow, and full. Head not so hot. There is a copious discharge of purulent mucus from the nose and ear.

Continue treatment.

Evening. Says he feels better. Eye looks natural. Had a stool and urinary discharge this morning.

Continue.

27*th*. Appeared considerably better.

Continue treatment.

Evening. Feverish and restless. No operation.

28*th*. No movement of the bowels for the last forty-eight hours. Gave an aloetic enema, which was followed by an evacuation.

Continue the iod. potass.

On the 30th the case was discharged from treatment, with directions to keep the bowels open. He took for five days 3 grs. iod. potass. every three hours. The daily record of symptoms is imperfect, as not conveying a due sense of the gravity of the symptoms.

Case III.—Edwin S., aged 2 years. Feb. 6th, was called to see him. Up to Feb. 15th, the symptoms were of a shifting febrile character, with cough, hurried respiration, some stupor, and occasional intolerance of light.

On the 15th he had symptoms of bronchitis, with a tight ringing cough. Some expectorants were given. On the 16th there was an extremely profuse secretion of mucus in the lungs, in such quantity as to materially impede respiration. He had a profuse perspiration, and also a diarrhœa; was pale and exhausted, and had hardly strength enough to free his lungs of the constantly rising mucus.

The treatment consisted of carb. ammon., quinine, and other supports. Under these he improved until the 18th, when he had an accession of fever, with two well-marked dysenteric discharges. For this (although he was very drowsy) opiates were given. He had, all this time, only such head symptoms as might be accounted for by the irritations elsewhere. The dysentery was checked by the opiates.

20*th*. Morning. Head symptoms more marked; pupils somewhat contracted; head hot. He screams occasionally with a very shrill voice—*cri hydrencephalique.*

℞ Potass. nitras, gr. ij, every two hours.

3, P. M. Head symptoms are increasing. Pupils of the eyes very much contracted; head very hot; respiration labored and interrupted. There is general coma, and the pupil is entirely insensible to light.

℞ Iod. potass., grs. v. every three hours. Ice bladder to the head, and warmth to the feet.

21*st*. Still insensible; chin quivers; pulse unsteady in volume, but cannot be called intermittent. Pupils of the eyes no larger than last night. Some diarrhœa.

Continue treatment.

Evening. Pulse slower and steady; head cooler; opens his eyes, and recognizes those around, apparently. The pupil of the right eye is very much enlarged. That of the left eye is enlarged, but is not more than one-half the size of the right.

Reduce the dose of iod. potass. to 3 grs.

Continue the revulsives.

22*d*. Has his senses; skin cool; head getting quite cool. In the right eye the pupil is so much dilated that a mere rim of the iris is visible.

℞ Emp. vesicat. to the nuchæ.

Continue the previous treatment.

23*d*. My patient is extremely cross and fretful, but has quite an appetite. and appears every way better. Objects to the ice bladder for the first time; pupils less dilated, and nearer of a size.

Omit the ice. Continue the iodide.

Evening. Pupils answer in some degree to the light; are both still enlarged, but nearer of a size, the right one having decreased, while the left remained stationary.

On the 24th I considered him convalescent. He was for some time extremely peevish and irritable, and two months elapsed before the iris regained its natural contractibility.

The iodide was continued, in smaller doses, for some time. The record gives 112 grs. iodid. potass. as consumed in four days. As is usual in bedside pharmacy, the quantity was guessed at, and was really much larger than is stated, as I subsequently ascertained by weighing a bulk equal to that consumed during this period. In all these cases the large dose was tolerated by the stomach, and I discovered uo unpleasant effects from it.

I have published only these three cases, because I have no doubt about the fact of effusion having occurred in them. In other cases I have prescribed the iodid. potass. for apparent cerebral disease, but as some doubt exists upon their true pathology, I do not instance them.

In Case I the subsequent loss of power of locomotion, and in Case II the continued paralysis of the iris, were subsequent symptoms, which confirm my previous diagnosis. The rationale of the remedy is simple.

I have long considered the iodide of potassium as a most serviceable diuretic, while its acknowledged action of stimulating the absorbents, peculiarly qualifies it for serous effusion. In cases of cerebral disease, we are usually unable to check them before the period of effusion, which sometimes comes on with astonishing rapidity. Those medicines—*e. g.* calomel—given with the view of controlling the inflammatory action, have not sufficient time to reach the difficulty, even if we admit that they have the power to control it. The use of drastic cathartics, so long and so strenuously urged, is, to my sense, not only unphilosophical, but injurious. The irritation of the alimentary mucous membrane is added to existing difficulties, and complicates, without relieving the case.

In an inflamed brain every irritation is felt with more than ordinary severity. It should be a maxim in these cases, to avoid unnecessarily distressing the patient. Consequently, blisters are less serviceable than in effusions elsewhere, and though the draft upon the fluids, which they occasion, is desirable, they should be avoided until the effusion is evident, and the capacity of pain in the cutaneous nerves is obtunded by the loss of cerebral sensibility.

To the iodide of potassium none of these objections apply, for no pain or irritation follows its use.

The theory of simple hydrocephalus would seem to be comprised in the following indications:

1. To combat the local inflammation by cold to the head, and the warm bath.

2. To promote the action of the kidneys, and thus to hasten the absorption of effused fluid.

3. To maintain a soluble condition of the bowels without the use of drastic or irritating medicines. For this purpose I prefer the aloetic enema, attributing to it a revulsive, as well as cathartic effect.

4. To counter-irritate and set up artificial discharges of serum.

While I have a reasonable conviction of the real efficacy of the iodide of potassium in simple hydrocephalus, the question must remain unsettled until a large number of cases is obtained. I am, however, convinced that should it be proved that it exerts no influence upon the disease, it will be found, also, that the previous modes of treatment are objectionable on other and stronger grounds, and treatment will be finally limited to the employment of external revulsives only.

